# Intraocular Pressure Changes of Healthy Lowlanders at Different Altitude Levels: A Systematic Review and Meta-Analysis

**DOI:** 10.3389/fphys.2019.01366

**Published:** 2019-11-06

**Authors:** Yiquan Yang, Yuan Xie, Yunxiao Sun, Kai Cao, Shuning Li, Sujie Fan, Lu Huang, Shizheng Wu, Ningli Wang

**Affiliations:** ^1^Beijing Key Laboratory of Ophthalmology and Visual Sciences, Beijing Tongren Eye Center, Beijing Institute of Ophthalmology, Beijing Tongren Hospital of Capital Medical University, Beijing, China; ^2^Department of Ophthalmology, The Third Hospital of Handan (Handan City Eye Hospital), Handan, China; ^3^Department of Neurology, The Qinghai Provincial People's Hospital, Xining, China

**Keywords:** intraocular pressure, high altitude, hypoxia, duration, exposure

## Abstract

**Background:** High altitude, characterized by hypobaric hypoxia, low temperature, and intensive ultraviolet radiation, is identifiably one of the examples of scientific enquiry into aviation and space analogs. However, little is known about the ocular physiological response, especially intraocular pressure (IOP) changes at high altitude.

**Objectives:** This study aimed to systematically review of high altitude exposure on IOP for healthy lowlanders with unoperated eyes.

**Methods:** A comprehensive systematic literature search was conducted in the electronic databases until September 1st, 2019. A meta-analysis was performed following the preferred reporting items for systematic review and meta-analysis statement (PRISMA). We systematically searched the studies conducted over 2,000 m above sea level (a.s.l) in healthy lowlanders with measurements of IOP. Meta-analyses (random effect model and heterogeneity tests), subgroup analyses (altitude, duration, type, and pattern of exposure), sensitivity analysis, funnel plot, Begger's and Egger's test for publication bias were performed. Quality assessment was conducted using the Newcastle–Ottawa scale. The meta-analysis was registered in the PROSPERO database (CRD42019136865).

**Results:** Of 9595 publications searched, 20 publications (*n* = 745) qualified for inclusion, with non-significant decrease in overall IOP [standardized mean difference (SMD): 0.14, 95% CI: −0.12–0.40; *p* = 0.30] with high heterogeneity (*p* < 0.001, *I*^2^ = 82%). However, subgroup analyses revealed significant decrease of IOP at high altitude of 3,000–5,500 m a.s.l (SMD: 0.57, 95% CI: 0.07–1.06; *p* = 0.03) whereas increase of IOP at extreme altitude of over 5,500 m a.s.l (SMD: −0.34, 95% CI: −0.61–0.06; *p* = 0.02). And the duration of exposure more than 72 hours (h) was likely to induce a decrease of IOP bordering on statistical significance at the 5% level (SMD: 1.29, 95% CI: 0.02–2.56; *p* = 0.05). Simultaneously, we also observed significant decrease of IOP for active exposure (e.g., physical activity and hiking, SMD: 0.81, 95% CI: 0.05–1.57; *p* = 0.04).

**Conclusion:** Our analysis shows exposure to the altitude over 3,500 m a.s.l, duration of exposure more than 72 h and active exposure pattern may have modest, but significant effects on IOP. The higher altitude, the duration of exposure as well as physical activity seem to play crucial roles in the effects of high-altitude exposure on IOP.

## Introduction

### Rationale

With the rapid development of economy, increasing numbers of lowlanders are traveling to high altitude for work, study, or pleasure. Given the prevalence of medical conditions in the general population, it is likely that many of these travelers will have one or more medical problems (Swenson and Bärtsch, 2014). In general, exposure to the elevation over 1,500 m a.s.l starts to have effects on human body. Short- as well as long-term exposure to high altitude environments over 2,400 m a.s.l causes physiological and pathological changes such as acute mountain sickness (AMS), high-altitude cerebral edema (HACE), or high-altitude pulmonary edema (HAPE) (Cymerman and Rock, 1994; Swenson and Bärtsch, 2014). Based on the effects of altitude and acclimatization on performance and well-being in healthy individuals, Bärtsch and Saltin (2008) proposed the following definition of altitudes, in which they divided highland into near sea (0–500 m a.s.l), low altitude (500–2,000 m a.s.l), moderate altitude (2,000–3,000 m a.s.l), high altitude (3,000–5,500 m a.s.l), and extreme altitude (>5,500 m a.s.l).

Notably, exposure to naturally high altitude environments has been also shown effects on eyes. The long-term exposure causes a number of eye disorders, such as pterygium (Lu et al., 2010), dry eye (Gupta et al., 2008), and lens opacity (Brilliant et al., 1983), whereas the short-term exposure often causes changes in visual function (Willmann et al., 2010; Gibson and Mckenna, 2011), refractive error (Mader et al., 1996), cornea thickness (Morris et al., 2007), retina vessels (Liu et al., 2013; Yang et al., 2019), optic nerve (Bosch et al., 2008; Schatz et al., 2018), as well as IOP.

In 1918, Wilmer and Berens (1918) first focused on the IOP changes at high altitude and measured IOP in 14 airmen using hypobaric chamber, but they did not detect any significant IOP changes. Since then, numerous studies have reported the IOP changes during real high-altitude exposure or simulated hypobaric hypoxic exposure for nearly one century. However, the conclusions of previous studies are not consistent, and even conflicting, reporting an increase, decrease, or no change. These inconsistencies are unclear and may be partly explained by the discrepancies in the elevation at which the studies was conducted, the approaches of ascending to the higher altitude, the different types of hypoxia exposure (rapid vs. slow and gradual ascent), the duration of high-altitude exposure, failure of most studies to correct for changes in corneal thickness, differences in IOP measurement techniques and individual susceptibility.

### Objectives

To date, the IOP changes at different altitude levels among healthy lowlanders have not been well-described. Consequently, we conducted this meta-analysis and to systematic review determine the effect of high altitude exposure on IOP for healthy lowlanders and to elaborate possible mechanisms comprehensively.

## Methods

### Systematic Review Protocol

The meta-analysis was conducted following the preferred reporting items for systematic review and meta-analysis statement (PRISMA) (Liberati et al., 2009) and was prospectively registered with the PROSPERO registry (CRD42019136865) in order to provide the highest level of quality.

### Search Strategy

A systematic literature search was performed using the following electronic databases: PubMed, Embase, and Cochrane Central Register of Controlled Trials (CENTRAL), China National Knowledge Infrastructure (CNKI), and Wan-fang for all medical publications. Each database was searched from their inception up to September 1st, 2019. The search strategy used key words and specific thesaurus terms (MeSH in Medline and EMTREE in Embase) and was systematically combined by the use of Boolean operators (AND/OR), which were detailed as: (“intraocular pressure”[MeSH Terms] OR (“intraocular”[All Fields] AND “pressure”[All Fields]) OR “intraocular pressure”[All Fields]) AND ((high[All Fields] AND (“altitude”[MeSH Terms] OR “altitude”[All Fields])) OR hypobaric[All Fields] OR (“hypoxia”[MeSH Terms] OR “hypoxia”[All Fields]) OR (low[All Fields] AND (“pressure”[MeSH Terms] OR “pressure”[All Fields]))). The searches were restricted to studies on human being, and language was restricted to English or Chinese. To supplement the online search, bibliographies of potentially relevant original publications, reviews, and meta-analysis were manually examined and screened for eligibility.

### Inclusion and Exclusion Criteria

Eligible studies were identified if they fulfilled the following criteria: (1) all participants were healthy sea-level natives and had no exposure over 500 m a.s.l in last 3 months before studies; (2) within-subject design was used; (3) studies conducted at simulated or real condition with elevation over 2,000 m a.s.l; (4) measurements of IOP with original data or other available data for the calculation of means and SDs; (5) language was restricted to English or Chinese only. We excluded studies that met the following criteria: (1) participants with prior ocular diseases, ocular surgeries and contact lens; (2) non-hypobaric hypoxic exposure; (3) the quality assessment score lower than 5; (4) studies published in animal studies, case reports, reviews, abstracts, commentaries conference proceedings, and editorials.

### Data Sources, Study Selection, and Data Extraction

Three of the authors selected studies according to the inclusion and exclusion criteria. Two independent reviewers (YY and XY) independently sifted through the titles and abstracts of articles obtained from search strategy and then examined to identify the final eligibility. When conflicts arose, disagreements were adjudicated by the third reviewer (YS). Two authors collected data following: (1) study information, including the authors, study design, the year of publication, sample size, age range, elevation of the baseline and summit, and ascent profile including chamber studies or field studies and exposure pattern, i.e., passive exposure (no physical activity or hiking) and active exposure (physical activity and hiking); (2) outcome measures of reported unadjusted and adjusted IOP; and (3) quality assessment of all the included studies, which appraised according to the guidelines for reporting meta-analysis of observational studies by using the Newcastle-Ottawa scale (Table 1). These checklists included selection (four items), comparability (two item), and outcome (three items). A study can be awarded a maximum of four stars for each item in the selection, two stars for comparability and three stars for outcome.

**Table 1:** Information abstracted from 19 eligible studies.

**References**	**Design**	**Population**	**Environment**	**Baseline/summit**	**Ascent profile**	**Outcomes**	**Tonometer**	**Quality assessment**
Najmanová et al. (2019)	Prospective	38 (male: 39.5%) Age: 25.2 ± 3.8 year	Hypobaric chamber	250 m/ 6,200 m (simulated)	Ascent rate:1,500 m/min	IOP and clinical parameters	I-Care Pro tonometer (Vantaa, Helsinki, Finland)	7
Albis-Donado et al. (2018)	Observational, cross-sectional	41 (male: 46.3%) Age: 41.7 ± 9.4 year	High altitude	Sea level (Mexico)/2,234 m (Mexico)	Sea level −2,234 m	IOP	Perkins tonometer	6
Willmann et al. (2017)	Prospective	14 (male: 50%) Age: 36 ± 9 year	High altitude	341 m (Germany)/4,559 m (Italy)	341 m −1,635 m −3,260 m −3,647 m −4,559 m	IOP, CCT, AMS score, and clinical parameters	Goldmann tonometer AT 900 (Haag-Streit BP 900, Haag-Streit, Koniz, Switzerland)	7
Baertschi et al. (2016)	Cohort, prospective	33 (male: 81.8%) Age: 46.4 ± 7.8 year	High altitude	300 m (Switzerland)/6,000 m (China)	300 m −4,200 m −6,000 m (trekking)	IOP, AMS score, and clinical parameters	I-Care tonometer (TAO1i, Helsinki, Finland)	6
Neumann et al. (2016)	Cohort, prospective	17 (male: 88.2%) Age: 36.7 ± 10.8 year	High altitude	140 m (Germany)/3,000 m (Switzerland)	140 m −1,800 m −3,000 m (by funicular)	IOP, retinal vessel diameter, AMS score, and clinical parameters	I-Care tonometer (TAO1i, Helsinki, Finland)	7
Nebbioso et al. (2014)	Prospective	20 (male: 100%) Age: 32 ± 5 year	Hypobaric chamber	Sea level (Italy)/5,486 m (simulated)	Ascent rate: 1,219 m/min(hypobaric chamber with 10% oxygen mask)	IOP, CCT	I-Care tonometer (TAO1i, Helsinki, Finland)	6
Jin et al. (2013)	Cohort, prospective	32 (male: 81.25%) Age: 28.97 ± 5.77 year	High altitude	200 m (China)/4,500 m (China)	200 m −2,500 m −4,500 m (by train)	IOP and clinical parameters	I-Care tonometer (TiolatOy, Finland)	8
Nazari et al. (2013)	Cohort, prospective	54 (male: 59.3%) Age: 35.78 ± 11.85 year	High altitude	1,900 m (Iran)/3,740 m (Iran)	Ascent rate: 61.3 m/min by gondola lift	IOP, AMS score, pulse rate, and arterial oxygen tension	Tono-Pen XL tonometer (Reichart Technologies,NY, USA)	7
Karakucuk et al. (2012)	Cohort, prospective	40 (male: 65%) Age: 15–49 year	High altitude	1,080 m (Turkey)/2,800 m (Turkey)	1,080 m −2,200 m (by bus) −2,800 m (trekking)	IOP, CCT, oxidation/antioxidation, and clinical parameters	Tono-Pen XL tonometer (Medtronic Solon, Jacksonville, FL)	6
Karadag et al. (2010)	Prospective	26 (male: 100%) Age: 23.1 ± 1.6 year	Hypobaric chamber	792 m (Turkey)/9,144 m (simulated)	Ascent rate: 417.6 m/min	IOP, arterial blood oxygen tension, and BNP	Tono-Pen XL tonometer (Medtronic-Solan, Jacksonville, USA)	6
Bosch et al. (2010)	Prospective, observational cohort	13 Age: 42 ± 12 year	High altitude	490 m (Switzerland)/6,265 m (China)	Ascent rate: 190 −200 m/d	IOP, AMS score, oxygen saturation, and optic disc appearance	Tono-Pen XL tonometer (Reichert, Inc., Depew, NY, USA)	6
Bosch et al. (2010)	Cohort, prospective	12 Age: 45 ± 9 year	High altitude	490 m (Switzerland)/6,265 m (China)	Ascent rate: 190 −200 m/d	IOP, AMS score, oxygen saturation, and optic disc appearance	Tono-Pen XL tonometer (Reichert, Inc., Depew, NY, USA)	6
Karadag et al. (2008)	Prospective	30 (male: 100%) Age: 24.8 ± 4.7 year	Hypobaric chamber	792 m (Turkey)/9,144 m (simulated)	Ascent rate: 417.6 m/min (hypobaric chamber with 100% oxygen mask)	IOP, CCT, and arterial oxygen saturation	Tono-Pen XL tonometer(Medtronic-Solan, Jacksonville, FL, USA)	6
Bayer et al. (2008)	Prospective	25 (male: 76%) Age: 34.9 ± 4.8 year	Air flight	536 m (Turkey)/2,438 m	536 m (baseline) −2,438 m	IOP	Tono-Pen XL tonometer (Mentor O&O, Norwell, MA, USA)	7
Somner et al. (2007)	Cohort, prospective	76 (male: 52.6%) Age: 22 ± 5 year	High altitude	Sea Level (United Kingdom)/5,200 m (Bolivia)	Sea level−3,700 m by plane−5,200 m (7 days)	IOP, CCT, AMS score, and blood pressure	Tono-Pen XL tonometer (Medtronic-Solan, Jacksonville, FL, USA)	6
Pavlidis et al. (2006)	Cohort, prospective	8 (male: 75%) Age: 37–67 year	High altitude	2,286 m (Pakistan)/5,050 m (Pakistan)	2,286 m −5,050 m by trekking	IOP, AMS score, and clinical parameters	Tono-Pen XL (Mentor O&O, Norwell, MA)	6
Ersanli et al. (2006)	Prospective	34 (male: 100%) Age: 31.9 ± 5 year	Hypobaric chamber	792 m (Turkey)/9,144 m (simulated)	Ascent rate: 417.6 m/min	IOP and clinical parameters	Tono-Pen XL tonometer(Medtronic-Solan, Jacksonville, FL, USA)	7
Bayer et al. (2004)	Cohort, prospective	20 (male: 90%) Age: 34.6 ± 9.5 year	Air flight	536 m (Turkey)/3,048 m (simulated)	Ascent rate: 251.2 m/min	IOP	Tono-Pen XL (Mentor O&O, Norwell, MA)	7
Cymerman et al. (2000)	Prospective	12 (male: 100%) Age: 29 ± 1 year	High altitude	50 m (America)/4,300 m (America)	50–1,835 m by plane −4,300 m by automobile	IOP and AMS score	Non-contact tonometer (CT-20 Tonometer, Topcon Corporation, Paramus, NJ)	7
Cymerman et al. (2000)	Prospective	7 (male: 100%) Age: 29 ± 1 year	Hypobaric chamber	50 m (America)/4,300 m (simulated)	Ascent rate: 354 m/min	IOP and AMS score	Non-contact tonometer (CT-20 Tonometer, Topcon Corporation, Paramus, NJ)	7
Cymerman et al. (2000)	Prospective	12 (male: 0%) Age: 26.1 ± 1.2 year	Hypobaric chamber	50 m (America)/4,300 m (simulated)	Ascent rate: 354 m/min	IOP and AMS score	Tono-Pen XL (Mentor O&O, Norwell, MA)	7
Clarke and Duff (1976)	Prospective	4	High altitude	1,500 m (Nepal)/5,400 m (Nepal)	1,500–3,000–5,400 m (24 days)	IOP and AMS score	Perkins tonometer	6
Newton et al. (1963)	Prospective	60 (male: 45%) Age: 20–37 year	Hypobaric chamber	Sea level/9,144 m (simulated)	Ascent rate: 609 m/min	IOP	Schiotz electronic tonometer (Mueller & Company, Chicago)	7

*IOP, intraocular pressure; CCT, central cornea thickness; AMS score, acute mountain sickness score*.

### Statistical Analysis

Meta-analysis was performed with Review Manager version 5.3 (The Nordic Cochrane Center, The Cochrane Collaboration, Copenhagen, Denmark) and Stata statistical software (v.14.0; Stata Corp, USA). Standardized mean difference (SMD) was used to make estimation of continuous variables, and weighted by inverse variance. The significance level was 0.05, 2-sided. Heterogeneity was evaluated using Chi-squared test and calculated by I square (*I*^2^) values, and the significant heterogeneity was assessed according to *p* < 0.10 or *I*^2^ more than 50%. Inverse variance random-effect models were adopted for pooling the SMD and 95% confidence interval (CI) for outcomes when heterogeneity was obvious. Subgroup differences analysis was done, and studies were split into three subgroups according to the altitude: moderate altitude (2,000–3,000 m a.s.l), high altitude (3,000–5,500 m a.s.l), and extreme altitude (>5,500 m a.s.l). We also divided studies into three subgroups by exposure duration: <12 h, 12–72 h, and 72 h-15 days (d). Exposure conditions were categorized as real high-altitude (field studies) and simulated exposure (field studies). Exposure patterns were categorized as active and passive exposure. Sensitivity analysis was performed to assess which study incurred undue weight in the analysis by the leave-one-out method. Potential publication bias were evaluated by the Begg's and Egger's funnel plot and a quantified result of *p* < 0.05 in Begg's and Egger's test indicated that publication bias existed.

### Quality Assessment

Two authors (YY and YX) independently extracted data and assessed the quality of included studies. Data were recorded on a customized data form. Discrepancies in data extraction and quality assessment were dealt with discussion.

## Results

### Study Selection and Characteristics

**Figure 1:**
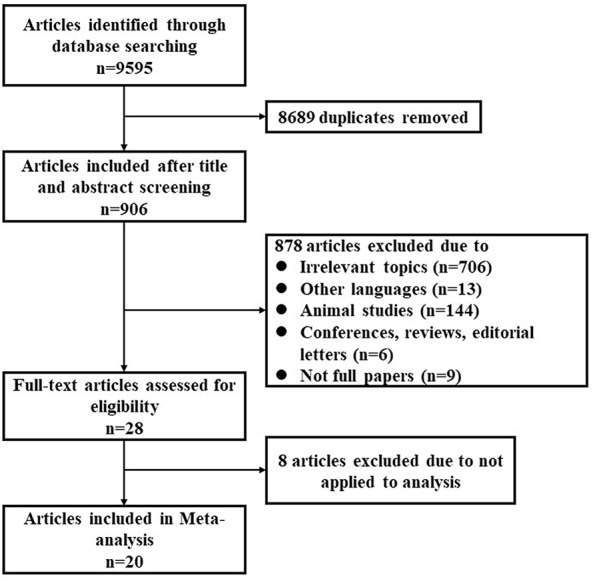
Flow chart representation of literature search.

Applying the search terms, a total of 9,595 publications were identified, of which 28 were considered relevant after screening by title and abstract (Figure 1). Taking inclusion and exclusion criteria into consideration, 23 studies from 20 full-text publications were selected for meta-analysis (Table 1). The ascent profiles differed with respect to altitude exposure as two studies were conducted during air flight, 13 studies during real high-altitude exposure, and eight under simulated conditions in hypobaric chambers (Table 1). Studies were divided into three groups according to exposure altitude: 2,000–3,000 m a.s.l, 3,000–5,500 m a.s.l, or >5,500 m a.s.l. Studies were divided into another three groups according to the duration of exposure: <12 h, 12–72 h, and 72 h-15 d. Furthermore, participants in eight studies were during active exposure whereas in 15 studies were during passive exposure in the ascent profiles. All of the listed publications were identified to be of good quality and the assessment reached six to eight stars.

### Effect of High-Altitude Exposure on IOP

**Figure 2:**
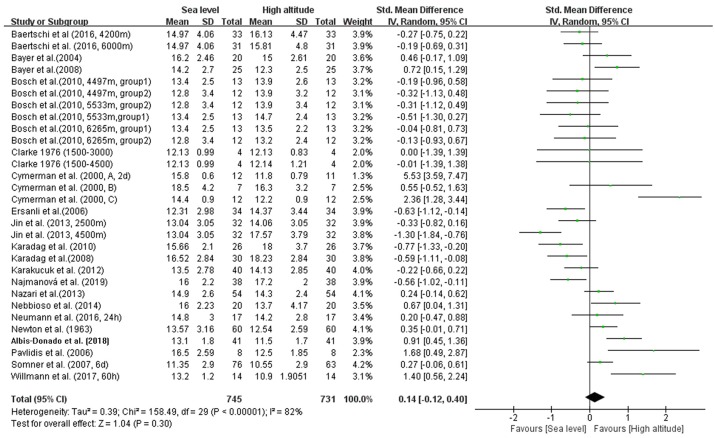
Forest plot for the meta-analysis of the effects of high-altitude exposure on IOP for healthy lowlanders. CI, confidence interval; df, degrees of freedom; G, group; IV, inverse variance; SD, standard deviation.

In our meta-analysis, the overall pooled differences between pre-exposure and post-exposure on IOP has been compared in 745 healthy lowlanders. Cumulatively, no significant difference were observed in IOP after high-altitude exposure (SMD = 0.14, 95% CI: −0.12–0.40, *p* = 0.30, Figure 2) with high heterogeneity (X^2^ test, *p* < 0.001, *I*^2^ = 82%).

### Subgroup Analysis

**Figure 3:**
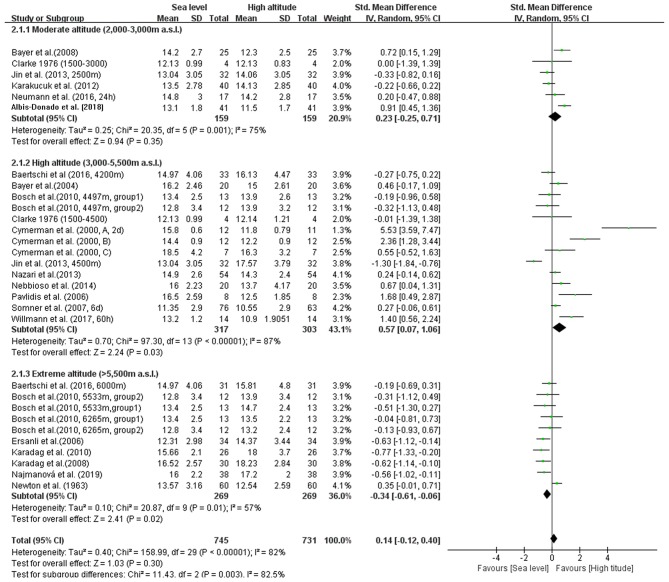
Forest plot of subgroup analysis at different altitude levels.

**Figure 4:**
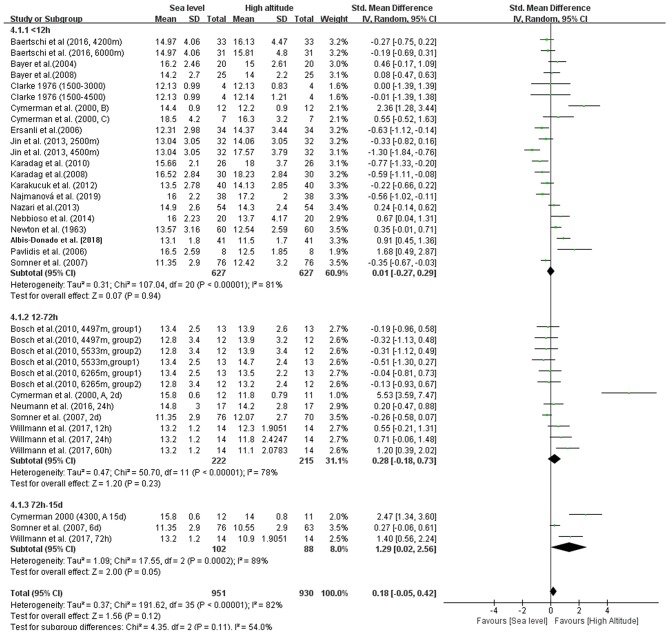
Forest plot of subgroup analysis for duration of high altitude exposure.

**Figure 5:**
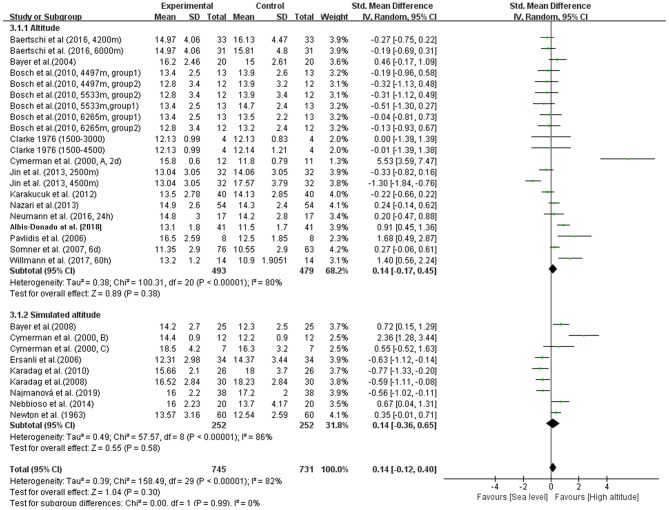
Forest plot of subgroup analysis for real and simulated altitude.

**Figure 6:**
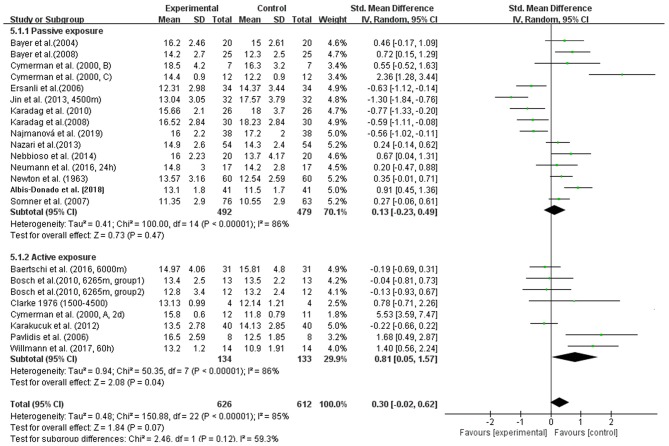
Forest plot of subgroup analysis for passive and active exposure to high altitude.

In addition, we conducted four subgroup analyses according to different elevations, duration of exposure, exposure conditions and patterns during ascent profiles (Figures 3–6). The results indicated significantly decreased IOP when trials were conducted at high altitude (SMD = 0.57, 95% CI: 0.07–1.06, *p* = 0.03) but significantly increased IOP when studies were conducted at extreme altitude (SMD = −0.34, 95% CI: −0.61 to −0.06, *p* = 0.02). Besides, the relatively long-term exposure (>72 h) may induce the decline of IOP which bordered on statistical significance at the 5% (SMD = 1.29, 95% CI: 0.02–2.56, *p* = 0.05). In contrast, no change of IOP was observed in real high-altitude exposure (SMD = 0.14, 95% CI: −0.17–0.45, *p* = 0.38) or simulated exposure (SMD = 0.14, 95% CI = −0.12–0.40, *p* = 0.58). Despite that, active exposure, such as physical activity, hiking, trekking, climbing in the field studies or bicycle exercise in the camber, could induce significantly decreased IOP for participants (SMD = 0.81, 95% CI: 0.05–1.57, *p* = 0.04). These subgroup analyses still demonstrated moderate to high heterogeneity ranging from 57 to 89%.

### Sensitivity Analysis and Publication Bias

**Figure 7:**
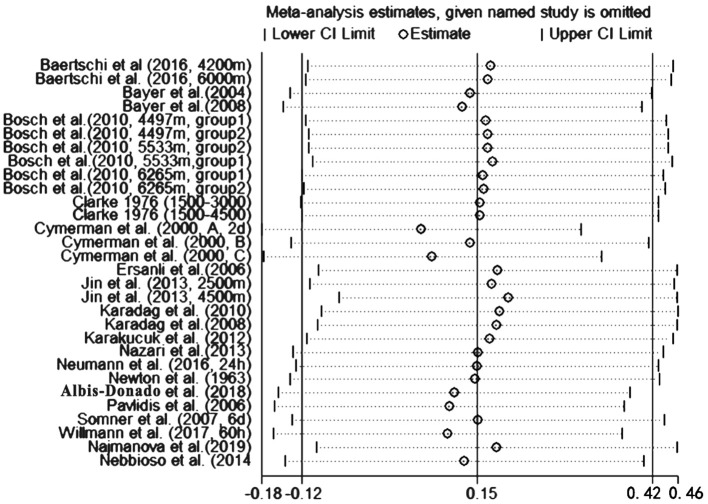
Sensitivity analysis changes in IOP during high altitude exposure.

**Figure 8:**
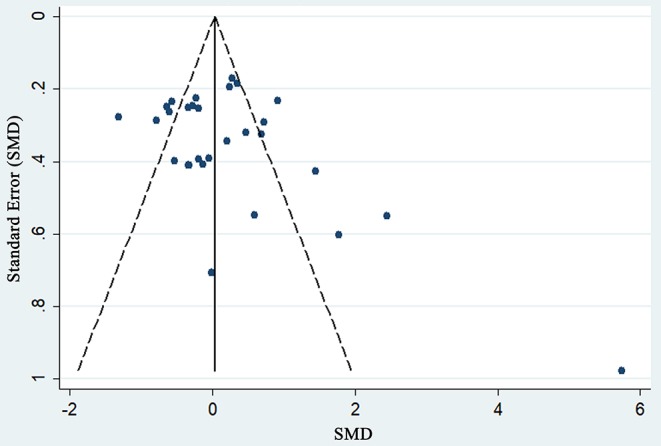
Funnel plot of changes in IOP during high altitude exposure.

To assess the stability of the meta-analysis, a sensitivity analysis was performed by recalculating pooled IOP level again when studies were successively eliminated one-by-one. Figure 7 shows that the corresponding pooled IOP level varied from 0.07 (−0.18–0.31) (excluding Cymerman et al., 2000, A, 2 d) to 0.20 (−0.06–0.46) (excluding Jin et al., 2013, 4,500 m a.s.l). The statistically similar results did not influence the stability and liability of the overall IOP level estimate in this meta-analysis. A funnel plot illustrating publication bias is shown in Figure 8. The distribution of the points was relatively asymmetric with one point located in far right corner, which may indicate an association with publication bias. Therefore, the overall publication bias was probably subsistent. However, the results of the Begg's test (*p* = 0.143) and the Egger's test (*p* = 0.120) demonstrated no evidence of significant publication bias. In general, there may be a small publication bias.

## Discussion

In current systematic review and meta-analysis, we analyzed data of 745 participants from 23 prospective studies of 20 publications, which indicated that high-altitude exposure induces a non-significant IOP decreasing. We conducted subgroup analysis of different elevations and demonstrated that this elevation-related effect requires high altitude (3,000–5,500 m a.s.l) for IOP decreasing and extreme altitude (>5,500 m a.s.l) for IOP increasing. Furthermore, the duration of exposure more than 72 hours (h) was likely to induce a decrease of IOP bordering on statistical significance at the 5% level and significant decrease of IOP for active exposure was observed.

Altitude-related illnesses are frequent causes of morbidity and occasional mortality in travelers to high altitude throughout the world (Imray et al., 2010). Its negative impact has also been observed in ocular conditions, and these effects are frequently encountered by mountaineers around the world (Karakucuk and Mirza, 2000). Skiers, sky-divers, paragliders, balloon travel enthusiasts are also at risk of developing AMS-like symptoms. IOP changes at high altitude have always been the subject of controversy. Obvious disagreements between studies illustrate the necessity of conducting an exhaustive review and quantitative analysis on all available evidence to determine the association between IOP and high-altitude exposure.

When it comes to altitude, we first consider the degree of altitude may have different impacts on IOP. Generally, higher altitude will lead to more severe hypoxia. Bosch et al. (2010) found IOP increasing up to an elevation of 5,533 m a.s.l. followed by a decrease with further ascent to 6,265 m a.s.l. during an expedition to Muztagh Ata (7,546 m a.s.l), which was consistent with our results. Interestingly, at the same time, the arterial oxygen saturation of altitude hikers also decreased to their lowest point at this elevation, which may suggest that the production of aqueous humor was suppressed by the depletion of oxygen in non-pigmented ciliary epithelium. Aqueous humor formation dysfunction caused by high-altitude hypoxia played an important role in IOP changes (Bosch et al., 2009, 2010; Nebbioso et al., 2014).

An additional essential factor is the acclimatization process. We also found that time-related effect requires >72 h of continuous high-altitude exposure to reach clinical significance in our meta-analysis. Generally, the degree of altitude acclimatization developed is proportional to the altitude reached and the duration of exposure. Bosch et al. (2010) also found that IOP reduction over time did occur, and a significant negative correlation between acclimatization time and IOP measurements was obtained. The fluctuation curve of IOP in Pavlidis' trial during climbing is more complete, the IOP showed a trend of decreasing along with the elevation rising. But in the acclimatization process, IOP has been somewhat recovered (Ersanli et al., 2006). The acclimatization process of IOP may be reflection of the acclimatization process of systemic oxygen saturation.

The results of our meta-analysis were mainly obtained by measuring the trekkers or hikers during climbing up to high altitude and the pilots or healthy subjects with simulated high altitude in the hypobaric hypoxia chamber. Compared to altitude hikers in real high altitude, individuals in hypobaric hypoxia chamber are lack of the influences of cold air, physical activity and tension. But our results showed that there was no difference for IOP changes during real or simulated hypobaric hypoxia chamber. The meta-analysis showed that active exposure to high altitude, such as physical activity, hiking, trekking, climbing and in the field studies or bicycle exercise in the camber, could induce significantly decreased IOP for participants. As we know, physical activities at high altitude has a decreasing effect on the IOP by reducing episcleral venous pressure (Lempert et al., 1967; Passo et al., 1987). So the exposure pattern accounts for the IOP changes at high altitude.

IOP readings are largely influenced by corneal properties especially corneal thickness. The cornea, under hypoxic conditions, undergoes a metabolic shift to anaerobic metabolism, which yields extracellular metabolic byproducts, causing a hydration pressure shift into the extracellular stromal spaces, leading to increased central corneal thickness (CCT) (Morris et al., 2007). The increased CCT may lead to an overestimate of IOP measurement at high altitude. However, only a few studies included had CCT measurement and IOP corrected after CCT. Furthermore, different types of tonometries were used to measure IOP changes in our meta-analysis. The applanation tonometer is supposed to be the gold standard in IOP measurement at high altitude because it is unresponsive to alterations in ambient barometric pressure (Neuburger et al., 2013; Willmann et al., 2017).

We speculate that mechanisms above are co-existence for healthy lowlanders from sea level to high altitude, but the factors affecting IOP occupy different positions at different altitudes. Further studies in real environments as well as in experimental settings are necessary to identify other potential risk factors for IOP at high altitude.

## Limitations

Potential limitations of our study should be considered. First, the leaders in the field and guideline in high altitude medicine, consistently define altitude at which lowlanders start getting symptoms above 2,500 m a.s.l. (Luks et al., 2017). The classification of altitude (above 2,000 m a.s.l.) is different from the elevation at which lowlanders start getting “altitude illness” symptoms. Second, it is noteworthy that heterogeneity in our meta-analysis was remarkably high. After carefully checking the studies included, we found that the elevation, duration and pattern of high-altitude exposure were different, and therefore subgroup analyses were performed. Third, 100% oxygen masks were provided to participants during ascent period for security of the two trials conducted in the hypobaric chamber. Then participants were performed measurement of IOP after removing their oxygen masks at the simulated target altitude. Hundred percentage oxygen inhalation during ascent period may have effect on IOP in the hypobaric chamber. Forth, IOP measurement is closely associated with the changes of CCT. IOP measurement errors may occur, and this was mainly because only a small proportion of studies included had CCT measurement at high altitude.

## Conclusions

In conclusion, the publications to date indicate that though no significant differences were observed in IOP after high-altitude exposure, subgroup analysis suggested exposure to elevation over 3,000 m a.s.l., duration of exposure >72 h may and active exposure pattern have modest, but statistically significant effects on IOP, which might be account for the inconsistent effects of high-altitude exposure on IOP. The degree of altitude reached, the duration of acclimatization process, physical activity, and the methodological quality of the studies were also determined to be potential sources of heterogeneity.

## Data Availability Statement

All datasets analyzed for this study are included in the manuscript and the supplementary files.

## Author Contributions

YY, YX, and YS conceived and designed the study. YY and YS involved in database search, extracted the data, and wrote the manuscript. KC analyzed the data. SL polished the English. SF, LH, SW, and NW revised the manuscript. All authors listed approved it for publication.

### Conflict of Interest

The authors declare that the research was conducted in the absence of any commercial or financial relationships that could be construed as a potential conflict of interest.
